# The Effect of Meals with Culinary Doses of Herbs and Spices on Postprandial Endothelial Function, Lipemia, and Glycemia after 4-Week Exposure to Herb- and Spice-Containing Diets: A Secondary Analysis of a Randomized, Crossover, Controlled-Feeding Study

**DOI:** 10.1016/j.cdnut.2026.109441

**Published:** 2026-07-21

**Authors:** Janhavi J Damani, Penny M Kris-Etherton, Connie J Rogers, David N Proctor, Kristin M Davis, Kristina S Petersen

**Affiliations:** 1 Department of Nutritional Sciences, Pennsylvania State University, University Park, PA, United States; 2 Department of Nutritional Sciences, University of Georgia, Athens, GA, United States; 3 Department of Kinesiology, Pennsylvania State University, University Park, PA, United States; 4 Department of Psychology, Wayne State University, Detroit, MI, United States

**Keywords:** herbs, spices, postprandial, flow-mediated dilation, hyperglycemia, lipemia, randomized clinical trial

## Abstract

**Background:**

Intake of herbs and spices improves postprandial responses; however, the acute effects of herbs and spices following longer-term exposure are unclear.

**Objectives:**

This secondary analysis aimed to examine the effect of meals containing 0.6 g (low), 3.7 g (moderate), and 7.4 g (high) of herbs and spices following intake of a diet with a similar daily amount of herbs and spices to the test meal on postprandial flow-mediated dilation (FMD), lipemia, and glycemia in adults at risk for cardiometabolic disease.

**Methods:**

The parent trial was a 3-period, randomized, crossover, controlled-feeding study. Participants consumed an average American diet containing low (0.5 g/2100 kcal/d), moderate (3.3 g/2100 kcal/d), and high (6.6 g/2100 kcal/d) doses of herbs and spices for 4 wk each (≥2-wk washout). At baseline and the end of each diet period, participants underwent a meal test (1192 kcal; 49% carbohydrates, 16% saturated fat). At baseline, the low-spice meal was provided, and at the end of each diet period, the meal contained herbs and spices at a dose that corresponded to that consumed during the preceding diet period. FMD (primary outcome) was measured at fasting and at 120 and 240 min after test meal intake. Triglycerides, glucose, and insulin were measured fasting and up to 240 min from test meal consumption. Between-condition effects were assessed using linear mixed-effect models.

**Results:**

The analytical sample included 43 adults (65% male; age: 48 ± 11 y; BMI: 28.9 ± 2.8 kg/m^2^). No between-condition differences were observed for FMD, triglycerides, glucose, and insulin (condition-by-time interaction, *P >* 0.05). The total area under the curve (AUC) for triglycerides and glucose did not differ between conditions. The insulin AUC was lower after the low-spice meal than the moderate-spice meal (mean fold difference: 0.89; 95% confidence interval: 0.82, 0.96, *P* = 0.011).

**Conclusions:**

After 4 weeks of herb and spice exposure, postprandial FMD, lipemia, and glycemia did not differ with acute intake of culinary doses of herbs and spices as part of a higher-carbohydrate, high-saturated-fat meal.

This trial is registered at clinicaltrials.gov as NCT03063320 (February 21, 2017; https://clinicaltrials.gov/study/NCT03063320).

## Introduction

Cardiometabolic diseases remain the leading cause of death in the United States [1], primarily attributed to poor diet quality [2]. Consumption of meals high in saturated fat and carbohydrates promotes postprandial hyperglycemia and hypertriglyceridemia [3,4], which better predict cardiovascular disease (CVD) risk than fasting measures [5,6]. Postprandial dysmetabolism is linked to oxidative stress, chronic inflammation, and endothelial dysfunction, an early independent predictor of CVD risk [7], which is widely assessed using flow-mediated dilation (FMD) [8]. Given the frequent consumption of high-saturated-fat, high-carbohydrate meals [9], strategies are needed to improve postprandial metabolic responses.

Herbs and spices are recommended as part of healthy dietary patterns to enhance flavor and reduce intake of sodium and added sugars [10]. Substantial evidence shows that individual herbs and spices, when consumed in high-dose capsule form, improve cardiometabolic risk factors [11]. Few trials have examined the postprandial effects of commonly consumed herbs and spices at culinary doses (amounts that can be feasibly consumed as part of dietary intake) [[12], [13], [14], [15], [16], [17], [18]]. Evidence from these studies demonstrates that consuming meals with higher culinary doses of herbs and spices (11–14.5 g/meal) improves postprandial lipemia [12,13], oxidative stress [14,15], and endothelial dysfunction [16] compared to meals without herbs and spices. Pilot research examining lower, more feasible doses of herbs and spices (2–6 g/meal) also shows attenuation of postprandial lipemia and endothelial dysfunction after intake of herbs and spices when consumed as part of high-fat, higher-carbohydrate meals compared to meals without herbs and spices [17,18]. Collectively, these findings suggest that culinary doses of mixed herbs and spices may acutely improve postprandial metabolic responses. It is unclear whether these postprandial metabolic benefits persist with longer-term consumption of herbs and spices.

We previously reported findings from a 3-period, randomized, crossover, controlled-feeding study in 71 adults at risk for cardiometabolic disease that showed that incorporation of a high culinary dose of mixed herbs and spices (6.6 g/2100 kcal/d) into an average American diet decreased 24-h blood pressure compared to lower culinary doses of mixed herbs and spices (0.5 and 3.3 g/2100 kcal/d) after 4 wk, without affecting fasting blood glucose, lipids/lipoproteins, or FMD [19]. It remains unclear whether intake of meals with culinary doses of mixed herbs and spices impacts postprandial responses after longer-term consumption of herbs and spices. The current study, nested within the parent trial [19], aimed to investigate the effect of meals containing 0.6 g (low), 3.7 g (moderate), and 7.4 g (high) of herbs and spices after intake of a diet with a similar daily amount of herbs and spices to the test meal on postprandial FMD and metabolic responses in adults at risk for cardiometabolic disease. It was hypothesized that incorporating culinary doses of herbs and spices into a high-saturated-fat, higher-carbohydrate meal would dose-dependently improve postprandial FMD and metabolic responses after longer-term consumption of herb- and spice-containing diets.

## Methods

### Trial protocol

The parent study [19] was a 3-period, crossover, controlled-feeding, randomized clinical trial designed to investigate the effect of consuming an average American diet (50% kcal from carbohydrates, 17% kcal from protein, and 33% kcal from total fat [11% saturated fat]; 3023 mg/2100 kcal/d sodium; 22 g/2100 kcal/d fiber) containing 3 doses of herbs and spices for 4 wk on cardiometabolic risk factors in adults at elevated risk for CVD (NCT03064932). The study design and primary findings have been described previously [19,20]. The current investigation focuses on the results of the postprandial meal challenge, which was nested within the parent trial [19]. The study protocol was approved by the institutional review board at The Pennsylvania State University. All participants provided written informed consent. This nested study is registered at clinicaltrials.gov (identifier NCT03063320).

### Participant eligibility

Eligible participants were aged 30 to 75 y, had a BMI (in kg/m^2^) of 25 to 35, elevated waist circumference (≥80 cm for females and ≥94 cm for males), and at least one of the following CVD risk factors: *1*) elevated glucose (100–126 mg/dL); *2*) elevated triglycerides (150–300 mg/dL); *3*) elevated LDL-cholesterol (>130 mg/dL); *4*) low HDL-cholesterol (<50 mg/dL for females and <40 mg/dL for males); *5*) elevated systolic and diastolic blood pressure (between 130/85 and 160/100 mmHg); or *6*) elevated high-sensitivity C-reactive protein (>1 mg/L). Only males and postmenopausal females were asked to complete the meal challenge to avoid confounding from hormonal variations associated with the menstrual cycle on FMD [8]. Participants were not eligible for inclusion if they currently or recently (≤6 mo) used tobacco products; were pregnant or lactating within the last 1 y; had allergies, intolerance, or aversions to study foods; were taking prescription medication, over-the-counter medication, or supplements known to affect study outcomes; had established CVD, stroke, cancer, diabetes, liver, kidney, or autoimmune diseases; had unstable body weight (>10% change within the last 6 months); or consumed >14 alcoholic beverages per week.

### Recruitment, screening, and randomization

Participants were recruited and enrolled between January 2017 and September 2019 from the State College, Pennsylvania area. Potentially eligible participants were screened at the Penn State Clinical Research Center to confirm eligibility. Prior to baseline testing, participants were randomly assigned to a diet sequence by a staff member (metabolic kitchen managers) not involved in participant recruitment and enrollment to enable allocation concealment. The randomization sequence was a computer-generated 6-sequence scheme that contained blocks of 6 sequences. All clinic staff and outcome assessors were blinded to the randomization. Although participants were unaware of their diet sequence, complete blinding was difficult to achieve because of potential differences in the taste characteristics of the test diets resulting from varying doses of herbs and spices.

### Intervention

Participants consumed 3 test diets that contained 24 different herbs and spices for 4 wk each: a low-spice diet (LSD) with 0.5 g/2100 kcal/d; a moderate-spice diet (MSD) with 3.3 g/2100 kcal/d; a high-spice diet (HSD) with 6.6 g/2100 kcal/d. Between each diet period, there was a ≥2-wk washout period. Adherence to the test diets was monitored by metabolic kitchen staff using self-reported daily checklists and was calculated as the proportion of study days on which participants reported consuming all provided study foods relative to the total number of prescribed study days, as previously described [19].

### Postprandial meal challenge

Meal challenges were conducted at baseline and at the end of each diet period, during which participants consumed a higher-carbohydrate, high-saturated-fat standardized test meal (1192 kcal; carbohydrate 145 g; protein 62 g; fat 44 g; saturated fat 20 g). The test meal consisted of a chicken tikka masala and an apple pie yogurt parfait. The total dose of herbs and spices in each test meal approximately corresponded to the dose of herbs and spices incorporated into the test diet that was consumed for the previous 4 wk as follows: 0.6 g LSM after the LSD (0.5 g/2100 kcal/d), 3.7 g MSM after the MSD (3.3 g/2100 kcal/d), and 7.4 g HSM after the HSD (6.6 g/2100 kcal/d) (Table 1). At the baseline visit, the LSM (0.6 g) was given. The test meal included 9 of the 24 herbs and spices used in the 4-wk diets, with coriander, turmeric, ginger, cumin, paprika, and cinnamon comprising a majority of the total dose. These herbs and spices were selected because they are commonly consumed in the United States [19] and have been associated with improved cardiometabolic outcomes [11].

**TABLE 1:** Spice composition of the test meals^1^

Herbs and spices,^2^ (g/meal) (% of total dose)	Low-spice meal	Moderate-spice meal	High-spice meal
Coriander (19%)	0.116	0.698	1.397
Turmeric (16%)	0.098	0.588	1.176
Ginger (15%)	0.092	0.551	1.103
Cumin (15%)	0.092	0.551	1.103
Paprika (15%)	0.092	0.551	1.103
Cinnamon (14%)	0.086	0.515	1.029
Cardamom (3%)	0.018	0.110	0.221
Red pepper (3%)	0.018	0.110	0.221
Garlic (1%)	0.006	0.037	0.074
Total dose (g/meal)	0.619	3.712	7.424

1 Nutrient composition of the test meal (1192 kcal; % of total energy for carbohydrates, 49%; protein, 21%; total fat, 33%; saturated fat, 16%; fiber, 3 g/d; sodium, 1460 mg/day) was estimated using Food Processor (ESHA Research).

2 Spices were weighed with a balance accurate to 0.001 gram (Mettler Toledo).

### Outcome assessment

Brachial artery FMD was the primary outcome of this nested study, and serum total cholesterol, LDL-cholesterol, HDL-cholesterol, triglycerides, insulin, and glucose were secondary outcomes.

At baseline and at the end of each 4-wk diet period, testing was conducted on 2 consecutive days after a 12-h fast. On the second day of testing at each time point, the postprandial meal challenge was conducted (Supplemental Figure 1). In the fasting state, FMD was assessed, a catheter was positioned, and a blood sample was taken by trained research nurses. Participants were then provided with the test meal and asked to consume the meal within 15 min.

#### FMD

FMD was measured in the fasting state and at 120 and 240 min after test meal consumption. These time points were selected based on the hypothesized mechanisms by which herb and spice intake may affect FMD. Prior evidence suggests that phenolic compounds peak in plasma ∼2 h after meal intake [21], whereas triglycerides, a key determinant of postprandial FMD [22], peak ∼4 h after meal intake [6]. Therefore, it was hypothesized that the phenolic effects of herbs and spices would be captured at 120 min, and given the previously observed attenuation of postprandial triglycerides observed at 240 min with herb and spice intake [12,13], this effect would be captured at 240 min.

All FMD assessments were performed by a single sonographer. After participants rested in the supine position for 5 min in a darkened room, FMD was assessed with a 10-MHz linear array transducer using a GE Logiq e (General Electric Company) ultrasound imaging system. Continuous, longitudinal images of the brachial artery at 5 to 10 cm above the elbow on the right arm were recorded at 5 frames/s during baseline (1 min), occlusion (5 min), and post-deflation (2 min). A blood pressure cuff was placed on the forearm (distal to the target artery) and inflated to 250 mmHg using an automated device (D. E. Hokanson, Inc.) to induce occlusion of the brachial artery for 5 min. The brachial artery diameter within the region of interest was measured continuously throughout the recording by 2 trained scorers using automated edge detection software (Brachial Analyzer; MIA). The resting diameter of the brachial artery (mm) was defined as the average of the values measured from all images collected throughout the 1-min baseline recording period. Peak artery diameter (mm) was defined as the largest diameter recorded within the first 2 min of the post-deflation period. Peak artery dilation was calculated by subtracting the resting artery diameter from the peak artery diameter post-deflation. FMD (%) was calculated as the percentage change in the brachial artery diameter from resting (baseline) to peak during post-deflation, i.e., [(peak artery diameter − resting artery diameter)/resting artery diameter × 100]. The average of the FMD values calculated by the 2 trained scorers was used for analysis; if the FMD values of the 2 scorers differed by >2 percentage points, a third person scored the scan, and the average of the 2 FMD values within 2 percentage points was then used for analysis. Flow velocity was measured using duplex-pulsed Doppler with the ultrasound beam at 2 time points, i.e., at the beginning of the baseline recording period and immediately after cuff release. Blood flow (milliliter per minute) was calculated based on the average of 5 cardiac cycles at each time point using the following equation: velocity time integral × cross-sectional area of the blood vessel [π × (brachial artery diameter at baseline/2)^2^] × heart rate. Reactive hyperemia (%) was calculated as the percentage change in blood flow after cuff release and was calculated as [(peak blood flow − baseline blood flow)/baseline flow × 100].

#### Lipids, lipoproteins, glucose, and insulin

Blood was collected in the fasting state and at 30, 60, 120, 180, and 240 min post-meal consumption. Blood collected into serum separator tubes was left to clot for ∼30 min at room temperature and then centrifuged at 1590 × *g* (±90) for 15 min at room temperature. Blood collected in EDTA-coated plasma tubes was centrifuged immediately at the same setting. All fasting and postprandial plasma/serum aliquots were frozen at −80°C until analysis. Serum total cholesterol, LDL-cholesterol, HDL-cholesterol, triglycerides, and insulin and glucose from EDTA plasma were measured using a Cobas c311 Chemistry Analyzer (Roche Diagnostics) by the Biomarker Core Laboratory at Pennsylvania State University.

### Statistical analysis

The parent trial was powered for fasting LDL-cholesterol [19]. The primary outcome of this nested study was brachial artery FMD. An a priori power calculation showed that 35 participants would provide 80% power (α = 0.05) to detect a 0.9 ± 1.5% (mean ± SD) difference in FMD, based on findings from previous clinical trials [14,16] examining one or more of the spices used in the current study. All statistical analyses were performed using Statistical Analysis System (version 9.4; SAS Institute Inc). The normality of residuals was assessed using univariate analysis (PROC UNIVARIATE) and visual inspection of the residual distribution and normal probability (Q–Q) plots. Nonnormally distributed variables were log-transformed for analysis.

In the primary analysis, the between-condition difference in mean values for each outcome was assessed using linear mixed-effect models (PROC MIXED) by the presence of main effects for time, condition (LSM, MSM, or HSM), and the condition-by-time interaction. Participant nested within randomization sequence was modeled to account for the repeated-measures crossover design. Randomization sequence, condition, and time were modeled as fixed effects. For the primary analysis, if a condition or condition-by-time interaction was detected, post hoc pairwise comparisons were conducted, and the Tukey–Kramer method was used to control the family wise error rate.

As a secondary analysis, we evaluated whether postprandial FMD, triglycerides, insulin, and glucose after 4 wk of spice exposure differed from postprandial responses to the standardized test meal containing 0.6 g of herbs and spices consumed at the baseline visit (i.e., without prior 4-wk spice exposure) to assess the potential effect of adaptation to herb and spice exposure. Linear mixed-effects models (PROC MIXED) were used with fixed effects for time, condition (baseline LSM without 4-week spice exposure and LSM, MSM, and HSM after 4-week spice exposure), randomization sequence, and the condition-by-time interaction. Participant nested within randomization sequence was included as a random effect to account for the repeated-measures crossover design. A sensitivity analysis was conducted for glucose only to exclude data from one participant with an extreme spike in glucose (>200 mg/dL) at 30 min, and results remain unchanged (data not shown).

In another secondary analysis, the between-condition difference in the total area under the concentration–time curve (AUC), estimated using the trapezoidal method [23], for FMD, glucose, insulin, and triglycerides was assessed using linear mixed-effect models (PROC MIXED). Participant nested within randomization sequence was modeled to account for the repeated-measures crossover design. Randomization sequence and condition were modeled as fixed effects. The total AUC for the baseline visit where participants received the LSM was included in the model as a covariate. Study visit (1, 2, or 3) was included as a fixed effect in the model to assess the presence of carryover effects; when a visit-by-condition interaction was not detected, visit was removed from the model. Sex (male or female) was included as a fixed effect in the model to assess sex differences in condition response; when a sex-by-condition interaction was not detected, sex was removed from the model. No significant carryover or sex effects were detected.

In exploratory analyses, Pearson correlation coefficients (PROC CORR) were used to assess the relationship between the change in triglycerides and the change in FMD during the postprandial period. Correlations were assessed by time (change from fasting to 120 and 240 min) and by condition (baseline, LSM, MSM, HSM) separately to account for the crossover design. In addition, the variation in the total AUC for FMD, glucose, insulin, and triglycerides in response to the postprandial meal challenge attributable to baseline participant characteristics was quantified using multiple linear regression (PROC REG) for each condition separately (baseline, LSM, MSM, HSM). In these models, baseline participant characteristics, including age, sex, BMI, the presence of metabolic syndrome (MetS) [24] (no if <3 MetS criteria or yes if ≥3 MetS criteria), and fasting values for each outcome, were included. The partial *R*^2^ was reported for predictors identified in the model using stepwise, bidirectional regression. Between-person and within-person variation in the total AUC for FMD, glucose, insulin, and triglycerides were quantified using a linear mixed-effect model (PROC MIXED), with randomization sequence and condition (LSM, MSM, or HSM) modeled as fixed effects and participant nested within randomization sequence modeled to account for the repeated-measures crossover design. The total AUC for the baseline visit where participants received the LSM was not included in the model as a covariate. The participant-level random intercept represented between-person variance, and the residual term represented within-person variance. The intraclass correlation coefficient (ICC) was calculated as the random-intercept variance divided by total variance, indicating the proportion of variance attributable to between-person differences [25].

Selection of model covariance structures was based on optimizing fit statistics (evaluated as the lowest Bayesian information criterion). Data analyses included all randomly assigned participants with data available at ≥1 time point. The mixed-models procedure does not perform listwise deletion, preserving degrees of freedom; therefore, this analytic approach allows inclusion of participants with ≥1 missing data point. Descriptive data are presented as mean ± SD, unless otherwise stated. Linear mixed-effect model-based estimates are presented as least-squares mean ± SEM. Estimates for log-transformed variables are presented as geometric mean [95% confidence interval (CI)]. Data from post hoc testing are presented as the pairwise mean between-condition difference and 95% CI with the Tukey–Kramer adjusted *P* value to control for the familywise error rate across all pairwise comparisons within each outcome. For log-transformed variables, data from post hoc testing are presented as fold differences and 95% CI between conditions to reflect the multiplicative relationships in the log-transformed scale before back-transformation. Graphs were plotted using GraphPad Prism version 10.0 (GraphPad Software). Statistical significance was set at *P* < 0.05.

## Results

A complete description of participant flow and baseline characteristics of the participants included in the parent trial (*n* = 71) has been described elsewhere [19]. Participants demonstrated high adherence, self-reporting consumption of all provided study foods on 94% of the study days, with comparable adherence (93%–95%) across all 3 diet periods and diet conditions as previously described [19]. The analytical sample for this study included 43 adults (65% male) with a mean age of 48 ± 11 y, BMI of 28.9 ± 2.8, and waist circumference of 102 ± 6.8 cm (Table 2). The baseline characteristics of this sample by randomization sequence are presented in Supplemental Table 1.

**TABLE 2:** Baseline characteristics of participants in the analytical sample overall

Baseline characteristics	Overall (*n* = 43)
Sex, *n* (%)	
Female	15 (35)
Male	28 (65)
Age (y)	48 ± 11
Anthropometric measures	
Weight (kg)	88.4 ± 13.3
Body mass index (kg/m^2^)	28.9 ± 2.8
Waist circumference (cm)	102 ± 6.8
Female	99.5 ± 7.7
Male	103 ± 6.0
Blood biochemical measures (fasting) (mg/dL)	
Total cholesterol	191 ± 31
LDL-cholesterol	127 ± 26
HDL-cholesterol	48 ± 12
Triglycerides	103 ± 40
Insulin (μIU/mL)	10.3 ± 5.5
Glucose	99 ± 8
Brachial artery blood pressure (fasting) (mmHg)	
Systolic	130 ± 13
Diastolic	81 ± 10
MetS criteria at baseline	
≥3, *n* (%)	18 (41.9%)
Brachial artery endothelial function (fasting)	
Flow-mediated dilation (%)	6.2 ± 2.3

Data are presented as mean ± SD, unless otherwise stated.

Abbreviation: MetS metabolic syndrome.

**FIGURE 1:**
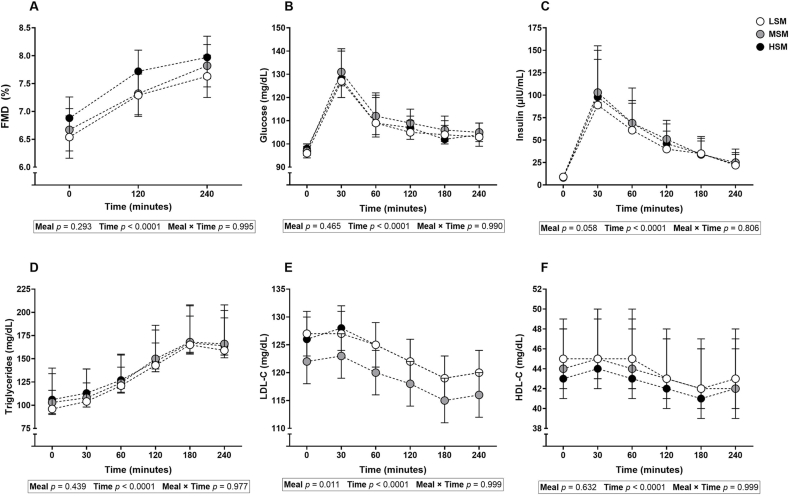
Mean values by condition and time point for (A) FMD (%); (B) glucose (mg/dL); (C) insulin (μIU/mL); (D) triglycerides (mg/dL); (E) LDL-cholesterol (mg/dL); (F) HDL-cholesterol (mg/dL) Data for FMD and LDL-cholesterol are presented as least-squares mean ± SEM. Data for triglycerides, glucose, insulin, and HDL-cholesterol are presented as geometric mean (95% confidence interval). PROC MIXED (SAS Institute) was used for analyses with participants nested within randomization sequence modeled as a repeated factor. FMD, flow-mediated dilation; HDL-cholesterol, high-density lipoprotein-cholesterol; HSM, acute effect of a high-spice meal after 4-wk exposure to a high-spice diet; LDL-cholesterol, low-density lipoprotein-cholesterol; LSM, acute effect of a low-spice meal after 4-wk exposure to a low-spice diet; MSM, acute effect of a moderate-spice meal after 4-wk exposure to a moderate-spice diet; SAS, Statistical Analysis System.

There was no main effect of condition or the condition-by-time interaction for mean FMD (Figure 1A) and other brachial artery endothelial function measures, including resting and peak diameter, peak dilation, resting and peak blood flow, and reactive hyperemia (Supplemental Table 2). A main effect of time was observed for mean FMD (*P* < 0.0001), where, regardless of the meal spice dose, FMD was higher at 120- and 240-min post-meal consumption than in the pre-meal fasting state (Figure 1A).

There was no condition-by-time interaction for plasma glucose, or serum insulin, triglycerides, total cholesterol, LDL-cholesterol, or HDL-cholesterol (Supplemental Table 3). However, a main effect of time (all *P <* 0.0001) was observed for triglycerides, glucose, insulin, LDL-cholesterol, HDL-cholesterol (Figure 1B–F), and total cholesterol (Supplemental Figure 2). For LDL-cholesterol, there was a main effect of condition (*P* = 0.011), and post hoc testing showed that this was because of a difference between the MSM and LSM, but this was attenuated to nonsignificance after adjustment for multiple comparisons (estimated mean between-condition difference: 4.5 mg/dL; 95% CI: −0.6, 9.6; Tukey–Kramer adjusted *P* = 0.095).

No between-condition difference was observed for the total AUC for FMD, glucose, and triglycerides (Table 3). A main effect of condition was observed for the total AUC for insulin (*P* = 0.019), which was attributable to a difference between the LSM and MSM (mean fold difference: 0.89; 95% CI: 0.82, 0.96; Tukey–Kramer adjusted *P* = 0.015). Baseline visit AUC values for FMD, glucose, insulin, and triglycerides were all significant covariates (*P* < 0.0001), but the results remained unchanged after adjustment (Table 3). In the secondary analysis examining potential adaptation by including baseline LSM as a condition, there was no condition-by-time interaction for FMD (Supplemental Figure 3A) or for triglycerides, glucose, or insulin (Supplemental Figure 3B–D). No correlations existed between the change in FMD and the change in triglycerides after the baseline LSM without prior spice exposure and LSM or MSM after 4-wk spice exposure of similar dose for either time point (Supplemental Table 4). At 120 min after consumption of the HSM after 4-wk exposure to the HSD, a nominally significant inverse correlation existed between the change in triglycerides and the change in FMD (*r* = −0.31; *P* = 0.049). However, this correlation was not observed 240 min after consumption of the HSM.

**TABLE 3:** Total AUC for flow-mediated dilation and blood glucose, insulin, and triglycerides by condition^1^

Total AUC	Baseline^2^	LSM	MSM	HSM	Between-condition mean difference 3		Condition main effect (unadjusted)^4^	Condition main effect (adjusted)^5^
					LSM vs. MSM	LSM vs. HSM	*P* value	*P* value
FMD (% × min)	1721 ± 81	1725 ± 70	1748 ± 70	1819 ± 71	21.2 (−122, 165)	80.8 (−64, 226)	0.276	0.392
Glucose (mg/dL × min)	26,588 ± 425	25,719 ± 463	26830 ± 467	26421 ± 471	1111 (−49, 2271)	701 (−468, 1871)	0.074	0.066
Insulin (μIU/mL × min)^6^	14,805 ± 1213	11,584 (10,998, 15,378)^a^	12,984 (12,293, 17,019)^b^	12,529 (11,916, 17,057)^ab^	0.89 (0.82, 0.96)	0.93 (0.85, 1.01)	0.019	0.025
Triglycerides (mg/dL × min)^6^	35,730 ± 2077	32,888 (31,128, 38,927)	34,022 (31,129, 42,278)	34,606 (32,018, 42,538)	0.96 (0.89, 1.04)	0.94 (0.87, 1.02)	0.303	0.373

Baseline, *n* = 43; LSM, *n* = 43; MSM, *n* = 42; HSM, *n* = 41.

LSM: acute effect of LSM after 4-week exposure to LSD.

MSM: acute effect of MSM after 4-week exposure to MSD.

HSM: acute effect of HSM after 4-week exposure to HSD.

Abbreviations: CI, confidence interval; FMD, flow-mediated dilation; HSD, high-spice diet; HSM, high-spice meal; LSD, low-spice diet; LSM, low-spice meal; MSD, moderate-spice diet; MSM, moderate-spice meal; SAS, Statistical Analysis System.

1 Data are least-squares mean ± SEM, unless otherwise stated. Total AUC is over 240 min. PROC MIXED (SAS Institute) was used for analyses with participants nested within randomization sequence modeled as a repeated factor. When a main effect of condition was detected, post hoc pairwise comparisons were conducted, and the Tukey–Kramer method was used to adjust for multiple comparisons. Means in the same row without a common letter differ, *P* < 0.05.

2 Exposure to LSM at baseline without prior 4-week spice exposure; data are arithmetic mean ± SEM.

3 Between-condition mean difference in the total AUC (95% confidence interval) with LSM as the reference; for log-transformed variables, the between-condition mean differences are presented as fold differences (95% confidence interval) to reflect the multiplicative relationships in the log-transformed scale before back-transformation.

4 Main effect of condition, unadjusted.

5 Main effect of condition adjusted for baseline visit AUC value.

6 Data are nonnormally distributed and presented as geometric mean (95% confidence interval).

There was substantial interindividual variability in postprandial responses for FMD (Supplemental Figure 4), lipemia, and glycemia (Supplemental Figures 5–7) across all conditions. The ICCs for total AUC were 0.638 for FMD, 0.457 for glucose, 0.841 for insulin, and 0.789 for triglycerides (Supplemental Table 5). At the baseline visit, prior to the 4-wk spice diets, participant characteristics explained 71.9% of the variability in the total AUC for FMD (adjusted *R*^2^ = 0.719; *P* < 0.001), mainly attributed to fasting FMD (54.76%), followed by age (7.02%), sex (6.61%), and MetS (3.57%) (Supplemental Table 6). However, after 4 wks of exposure to herb- and spice-containing diets, only fasting FMD remained a predictor across all meal conditions. For the total AUC for glucose, insulin, and triglycerides, participant age, sex, BMI, or MetS status did not explain any variation at the baseline visit or after 4 wks of the spice-containing diets (Supplemental Tables 7–9).

## Discussion

The acute effect of mixed herbs and spices on postprandial metabolic responses has been studied largely by comparing culinary doses of herbs and spices with no-spice conditions [11], without any preceding long-term exposure to herbs and spices. In the current study, incorporating herbs and spices into a high-saturated-fat, higher-carbohydrate meal did not differentially affect postprandial FMD, lipemia, or glycemia in adults at risk of cardiometabolic disease after consumption of diets containing herbs and spices for 4 wks.

The hypothesis that longer-term exposure to herbs and spices would improve postprandial endothelial function was based on evidence from clinical trials showing improved postprandial endothelial function after consumption of meals containing similar herbs and spices but in higher doses (11–14.5 g/meal) than control meals without herbs and spices [14,16]. Furthermore, our previous pilot work suggested that incorporation of 6 g of herbs and spices into a high-saturated-fat, higher-carbohydrate meal may attenuate postprandial impairment in FMD compared to an isocaloric macronutrient-matched meal without herbs and spices in men (*n = 13*) with elevated waist circumference [17]. However, in the present study, which included both males and females with elevated waist circumference, FMD did not differ with acute exposure to lower, more feasible culinary doses of herbs and spices (0.6–7.4 g/meal) after longer-term dietary exposure to herbs and spices, which may be explained by several factors. Our study participants had higher-than-expected mean fasting FMD (6.2%), indicating normal endothelial function [26], with limited potential for further improvement. Previous studies showing improved postprandial FMD with herb and spice intake included individuals with impaired endothelial function (∼5.3%–5.5%) [16,17], who may be more responsive to intervention. Second, it is possible that the herb and spice effect may have been delayed beyond the 4-h study period. Huang et al. [18] showed that a high-saturated-fat, high-carbohydrate meal with 6 g of Italian herbs or pumpkin spice improved FMD 24 h after the meal, with no differences seen at earlier time points. It is also therefore plausible that chronic exposure to herbs and spices prior to the meal tests may have reduced our ability to detect postprandial effects. Although postprandial responses at baseline (no prior herb and spice exposure) did not differ from postprandial responses after the 4-wk diet periods, we may have been underpowered to detect subtle effects. Finally, our meal test did not induce FMD impairment, FMD was higher at 120- and 240-min post-meal intake than in the fasting state, which is typically observed with high-saturated-fat, higher-carbohydrate meals [3]. This may be because our test meal had a lower saturated fat content (16% of total energy) than the meal used in prior research (33% of total energy) [17].

In the current study, triglycerides, glucose, and insulin increased over time, which was expected given the macronutrient composition of the test meal, but did not differ between the herb and spice doses. This may also have contributed to the lack of differences observed in FMD, as higher-fat, higher-carbohydrate meals impair postprandial endothelial function by increasing postprandial glucose, triglycerides, and free fatty acids, which promote oxidative stress, inflammation, and reduced nitric oxide bioavailability [4]. However, as previously reported, in this trial, postprandial plasma proinflammatory cytokines (IL-1β, IL-8, and TNF-α) were lower after consumption of the MSM than after consumption of the LSM [20]. This suggests some attenuation of the meal-induced inflammatory response with herbs and spices, but it may have been insufficient to affect endothelial function. In trials in which postprandial attenuation of lipemia with herbs and spices has been observed [12,13], higher doses of mixed herbs and spices (∼14 g/meal) were studied. McCrea et al. [13] conducted in vitro experiments showing that the herb and spice blend studied, which attenuated postprandial lipemia, inhibited pancreatic lipase and phospholipase A2, suggesting that reduced intestinal fat absorption and chylomicron formation may explain the reduction in postprandial lipemia. Collectively, our findings suggest that lower doses of herbs and spices that may be more feasibly incorporated into meals may be inadequate to overcome the metabolic perturbation induced by a high-saturated-fat, higher-carbohydrate meal.

In our study, the total AUC for insulin was lower after the LSM than after the MSM and the HSM, although the latter did not reach statistical significance. It is not clear why this finding was observed, and the lack of direct assessment of insulin sensitivity limits interpretation. In prior work, mixed herbs and spices (14 g/meal) incorporated in a high-saturated-fat, higher-carbohydrate meal attenuated the total AUC for insulin by 21% more than control meals without herbs and spices in healthy males with overweight [12]. Our study population was likely less insulin sensitive and therefore may have been less responsive to the effects of herbs and spices. Future studies should incorporate comprehensive mechanistic assessments, including insulin sensitivity, oxidative stress, inflammation, and endothelial signaling pathways, to better elucidate the physiological mechanisms underlying the effects of herbs and spices on postprandial endothelial function in response to high-saturated-fat, higher-carbohydrate meals.

In our study, at the baseline visit, participant characteristics explained substantial variability in postprandial FMD, but after 4 wks of exposure to spice-containing diets, only fasting FMD remained predictive, suggesting that the standardized diet reduced between-person variability. High ICCs were observed for postprandial insulin and triglycerides, suggesting that between-person differences, beyond the baseline characteristics we assessed, largely explained these responses. In contrast, postprandial glucose had a lower ICC and greater within-person variability, suggesting a stronger influence of person-specific factors such as meal context (sleep, previous meals, physical activity) [27]. Our findings are consistent with prior work [27] demonstrating that baseline participant characteristics, genetics, and gut microbiome composition explained a significant proportion of variance in postprandial lipemia, whereas meal context had a greater influence than demographics or habitual diet for postprandial glycemia. Overall, our study showed interindividual variability in postprandial responses even with high adherence to the spice-containing diets and well-controlled experimental conditions. This variability may have reduced our ability to detect small intervention effects.

A major strength of this randomized crossover trial is the controlled-feeding design and blinding of all personnel involved in data collection and analysis. However, this study is likely underpowered given that a smaller-than-expected effect was observed for FMD. Compared with the LSM, the HSM increased FMD by 0.39% (mean difference in total AUC over 240 min); although this difference did not reach statistical significance, it is expected to correspond to an estimated 4.7% risk reduction in cardiovascular events [28], which suggests the possibility of a small but clinically relevant effect. Accordingly, the absence of statistically significant differences in FMD and most secondary outcomes should be interpreted as a lack of evidence of an effect rather than evidence of no effect, as this nested study may have been underpowered to detect modest but clinically meaningful effects. The lack of a meal condition without herbs and spices is another limitation of this study, as all participants were exposed to herbs and spices acutely, which may have reduced our ability to detect postprandial improvements. Other limitations include the lack of direct assessment of markers of oxidative stress and endothelial signaling to provide mechanistic insights into the findings. Finally, given the large number of secondary outcomes analyzed and the lack of correction for multiplicity, risk of type I statistical error is increased, and the results should be interpreted with caution.

In conclusion, this study showed that after 4 wks of exposure to herb- and spice-containing diets, no differences in postprandial endothelial function, lipemia, and glycemia were observed with the incorporation of herbs and spices in a higher-carbohydrate, high-saturated-fat meal. This suggests that any acute effects of incorporating herbs and spices in a higher-carbohydrate, high-saturated-fat meal may be less apparent after longer-term herb and spice exposure.

## Author contributions

The authors’ responsibilities were as follows – KSP, CJR, DNP, PMK-E: designed the research (project conception and development of the overall research plan); KSP, KMD conducted the research and collected the data (hands-on conduct of the study and data collection); JJD, KSP performed statistical analysis and interpreted the data; JJD, KSP wrote the initial manuscript draft; and all authors: read and approved the final manuscript.

## Data availability

Data described in the manuscript, code book, and analytic code will be made available upon reasonable request pending application and approval.

## Declaration of Generative AI and AI-assisted technologies in the writing process

No AI was used.

## Funding

This trial was funded by the McCormick Science Institute and by the National Center for Advancing Translational Sciences, National Institutes of Health, through grant UL1 TR002014. The funding agency had no role in data collection, analysis, or interpretation of data; or in the writing or submission of the manuscript. The content is solely the responsibility of the authors and does not necessarily represent the official views of the funding agency.

## Conflict of interest

KSP is a member of the McCormick Science Institute’s Scientific Advisory Council. This conflict has been reviewed by The Pennsylvania State University’s Individual Conflict of Interest Committee and is currently being managed by the University. All other authors report no conflicts of interest.
