# Supplementary material for: The Effect of Meals with Culinary Doses of Herbs and Spices on Postprandial Endothelial Function, Lipemia, and Glycemia after 4-Week Exposure to Herb- and Spice-Containing Diets: A Secondary Analysis of a Randomized, Crossover, Controlled-Feeding Study
Source: Curr Dev Nutr. 2026 Jul 21;10(8):109441. doi: 10.1016/j.cdnut.2026.109441 (PMC13475433; doi:10.1016/j.cdnut.2026.109441)
Supplement: Supplementary file 1 [file mmc1.docx]

**The Effect of Meals with Culinary Doses of Herbs/Spices on Postprandial Endothelial Function, Lipemia and Glycemia Following 4-week Exposure to Herb/Spice-Containing Diets: A Secondary Analysis of A Randomized, Crossover, Controlled-Feeding Study**

Damani *et al*. Online Supplemental Material

# ONLINE SUPPLEMENTAL FILES

## Table of Contents

**Supplemental Figure 1.** Design of nested study with postprandial meal challenge ….…………3

**Supplemental Figure 2.** Mean values by condition and timepoint for total cholesterol (mg/dL)……………………………………………………………………………………………5

**Supplemental Figure 3.** Mean values at each timepoint for baseline condition (no prior spice exposure) and post 4-week spice exposure conditions for (**A**) flow-mediated dilation (FMD) (%); (**B**) triglycerides (mg/dL); (**C**) glucose (mg/dL); (**D**) insulin (mg/dL)…………………………..6

**Supplemental Figure 4.** Inter-individual variation in postprandial FMD...…………………….7

**Supplemental Figure 5.** Inter-individual variation in postprandial triglyceride response….…..8

**Supplemental Figure 6.** Inter-individual variation in postprandial glucose response………….9

**Supplemental Figure 7.** Inter-individual variation in postprandial insulin response.………….10

**Supplemental Table 1.** Baseline characteristics of participants in the analytical sample by randomization sequence…………………………………………………………………………11

**Supplemental Table 2.** Mean values for brachial artery endothelial function assessment by condition and timepoint…………………………………………………………………………13

**Supplemental Table 3.** Mean values for blood glucose, insulin, lipids, and lipoproteins by condition and timepoint…………………………………………………………………………14

**Supplemental Table 4.** Relationship between the change in FMD (%) and change in triglycerides (mg/dL) by condition and timepoint.…………………………………………….15

**Supplemental Table 5.** Between-person and within-person variance in the total AUC for FMD, blood glucose, insulin and triglycerides……………………………………………………….16

**Supplemental Table 6.** Multiple Linear Regression for Brachial Artery FMD: Total Area Under the Curve (AUC) (% × min)……………………………………………………………………………………………18

**Supplemental Table 7.** Multiple Linear Regression for Postprandial Glucose: Total Area Under the Curve (AUC) (mg/dL × min)…………………………………………………………………………………………….20

**Supplemental Table 8.** Multiple Linear Regression for Postprandial Insulin: Total Area Under the Curve (AUC) (µIU/mL × min) ………………………………………………………………………………………………….22

**Supplemental Table 9.** Multiple Linear Regression for Postprandial Triglycerides: Total Area Under the Curve (AUC) (mg/dL × min)………………………………………………………………….........................................24

**The Effect of Meals with Culinary Doses of Herbs/Spices on Postprandial Endothelial Function, Lipemia and Glycemia Following 4-week Exposure to Herb/Spice-Containing Diets: A Secondary Analysis of A Randomized, Crossover, Controlled-Feeding Study**

Damani *et al*. Online Supplemental Material

# Supplemental Figure 1. Design of nested study with postprandial meal challenge.

Participants consumed three test diets that contained herbs/spices for 4 weeks each: a low-dose spice diet (LSD) with 0.5 g/2100kcal/day; a moderate-dose spice diet (MSD) with 3.3 g/2100 kcal/day; a high-dose spice diet (HSD) with 6.6 g/2100 kcal/day, with at least a 2-week washout between each diet period. At baseline and at the end of each 4-week diet period, testing was conducted on two consecutive days after a 12-hour fast. On the second day of testing following each diet period, participants were provided with a postprandial meal challenge containing a blend of herbs/spices that corresponded to the dose of herbs/spices of the diet consumed for the previous 4 weeks as follows: 0.6 g low-spice meal after the LSD (0.5 g/2100 kcal/day), 3.7 g moderate-spice meal after the MSD (3.3 g/2100 kcal/day), and 7.4 g high-spice meal after the HSD (6.6 g/2100 kcal/day) At the baseline visit, the low-spice meal (0.6 g) was given. *Abbreviations*: FMD, flow-mediated dilation

**The Effect of Meals with Culinary Doses of Herbs/Spices on Postprandial Endothelial Function, Lipemia and Glycemia Following 4-week Exposure to Herb/Spice-Containing Diets: A Secondary Analysis of A Randomized, Crossover, Controlled-Feeding Study**

Damani *et al*. Online Supplemental Material

# Supplemental Figure 2. Mean values by condition and timepoint for total cholesterol (mg/dL)

Data are presented as least squares means ± SEM. PROC MIXED (SAS Institute, Cary, NC, USA) was used for analyses with participants nested within randomization sequence modeled as a repeated factor.

*Abbreviations*: LSM: acute effect of low-spice meal (LSM) after 4-week exposure to low-spice diet (LSD); MSM: acute effect of moderate-spice meal (MSM) after 4-week exposure to moderate-spice diet (MSD); HSM: acute effect of high-spice meal (HSM) after 4-week exposure to high-spice diet (HSD).

**The Effect of Meals with Culinary Doses of Herbs/Spices on Postprandial Endothelial Function, Lipemia and Glycemia Following 4-week Exposure to Herb/Spice-Containing Diets: A Secondary Analysis of A Randomized, Crossover, Controlled-Feeding Study**

Damani *et al*. Online Supplemental Material

# Supplemental Figure 3. Mean values at each timepoint for baseline condition (no prior spice exposure) and post 4-week spice exposure conditions for (A) flow-mediated dilation (FMD) (%); (B) triglycerides (mg/dL); (C) glucose (mg/dL); (D) insulin (mg/dL)

Data for FMD are presented as least squares means ± SEM. Data for triglycerides, glucose, and insulin are presented as geometric mean (95% confidence interval). PROC MIXED (SAS Institute, Cary, NC, USA) was used for analyses with participants nested within randomization sequence modeled as a repeated factor.

*Abbreviations*: Baseline: exposure to LSM at baseline without prior 4-week spice exposure; LSM: acute effect of low-spice meal (LSM) after 4-week exposure to low-spice diet; MSM: acute effect of moderate-spice meal (MSM) after 4-week exposure to moderate-spice diet; HSM: acute effect of high-spice meal (HSM) after 4-week exposure to high-spice diet

**The Effect of Meals with Culinary Doses of Herbs/Spices on Postprandial Endothelial Function, Lipemia and Glycemia Following 4-week Exposure to Herb/Spice-Containing Diets: A Secondary Analysis of A Randomized, Crossover, Controlled-Feeding Study**

Damani *et al*. Online Supplemental Material

# Supplemental Figure 4. Inter-individual variation in postprandial FMD.

**(A)** Low-Spice Meal at Baseline**; (B)** Low-Spice Meal after 4-week LSD; **(C)** Moderate-Spice Meal after 4-week MSD; and **(D)** High-Spice Meal after 4-week HSD

Each colored line represents an individual participant, with colors consistent for the same participant across all panels.

*Abbreviations*: FMD, flow-mediated dilation; low-spice diet (LSD); moderate-spice diet (MSD); high-spice diet (HSD)

**The Effect of Meals with Culinary Doses of Herbs/Spices on Postprandial Endothelial Function, Lipemia and Glycemia Following 4-week Exposure to Herb/Spice-Containing Diets: A Secondary Analysis of A Randomized, Crossover, Controlled-Feeding Study**

Damani *et al*. Online Supplemental Material

# Supplemental Figure 5. Inter-individual variation in postprandial triglyceride response.

**(A)** Low-Spice Meal at Baseline**; (B)** Low-Spice Meal after 4-week LSD; **(C)** Moderate-Spice Meal after 4-week MSD; and **(D)** High-Spice Meal after 4-week HSD

Each colored line represents an individual participant, with colors consistent for the same participant across all panels.

*Abbreviations*: low-spice diet (LSD); moderate-spice diet (MSD); high-spice diet (HSD)

**The Effect of Meals with Culinary Doses of Herbs/Spices on Postprandial Endothelial Function, Lipemia and Glycemia Following 4-week Exposure to Herb/Spice-Containing Diets: A Secondary Analysis of A Randomized, Crossover, Controlled-Feeding Study**

Damani *et al*. Online Supplemental Material

# Supplemental Figure 6. Inter-individual variation in postprandial glucose response.

**(A)** Low-Spice Meal at Baseline**; (B)** Low-Spice Meal after 4-week LSD; **(C)** Moderate-Spice Meal after 4-week MSD; and **(D)** High-Spice Meal after 4-week HSD

Each colored line represents an individual participant, with colors consistent for the same participant across all panels.

*Abbreviations*: low-spice diet (LSD); moderate-spice diet (MSD); high-spice diet (HSD)

**The Effect of Meals with Culinary Doses of Herbs/Spices on Postprandial Endothelial Function, Lipemia and Glycemia Following 4-week Exposure to Herb/Spice-Containing Diets: A Secondary Analysis of A Randomized, Crossover, Controlled-Feeding Study**

Damani *et al*. Online Supplemental Material

# Supplemental Figure 7. Inter-individual variation in postprandial insulin response.

**(A)** Low-Spice Meal at Baseline**; (B)** Low-Spice Meal after 4-week LSD; **(C)** Moderate-Spice Meal after 4-week MSD; and **(D)** High-Spice Meal after 4-week HSD

Each colored line represents an individual participant, with colors consistent for the same participant across all panels.

*Abbreviations*: low-spice diet (LSD); moderate-spice diet (MSD); high-spice diet (HSD)

**The Effect of Meals with Culinary Doses of Herbs/Spices on Postprandial Endothelial Function, Lipemia and Glycemia Following 4-week Exposure to Herb/Spice-Containing Diets: A Secondary Analysis of A Randomized, Crossover, Controlled-Feeding Study**

Damani *et al*. Online Supplemental Material

# Supplemental Table 1. Baseline characteristics of participants in the analytical sample by randomization sequence

| **Baseline Characteristics** | **LSM-MSM-HSM**  (*n*=8) | **LSM-HSM-MSM**  (*n*=10) | **MSM-LSM-HSM**  (*n*=8) | **MSM-HSM-LSM**  (*n*=4) | **HSM-LSM-MSM**  (*n*=7) | **HSM-MSM-LSM**  (*n*=6) |
| --- | --- | --- | --- | --- | --- | --- |
| **Sex,** *n* (%) |  |  |  |  |  |  |
| Female | 3 (38) | 4 (40) | 3 (38) | 1 (25) | 2 (29) | 2 (33) |
| Male | 5 (62) | 6 (60) | 5 (62) | 3 (75) | 5 (71) | 4 (67) |
| **Age,** years | 48 ± 12 | 49 ± 11 | 42 ± 12 | 43 ± 12 | 55 ± 10 | 50 ± 11 |
| **Anthropometric measures** | | | | | | |
| Weight, kg | 82.3 ± 6.8 | 92.1 ± 14.9 | 90.8 ± 15.1 | 88.5 ± 18.1 | 85.0 ± 12.9 | 91.1 ± 13.6 |
| Body mass index, kg/m^2^ | 28.1 ± 2.4 | 29.3 ± 3.2 | 28.8 ±2.6 | 28.2 ± 3.1 | 28.2 ± 2.9 | 30.6 ± 3.2 |
| Waist circumference, cm | 100 ± 4.6 | 103 ± 6.9 | 100 ± 6.4 | 100 ± 7.7 | 101 ± 9.0 | 106 ± 6.3 |
| Female | 103 ± 6.2 | 101 ± 8.9 | 98 ± 8.8 | 93 ± 7.7 | 93 ± 4.9 | 104 ± 10.6 |
| Male | 98 ± 2.3 | 104 ± 5.7 | 100 ± 5.6 | 103 ± 7.0 | 105 ± 8.0 | 107 ± 4.6 |
| **Blood biochemical measures (fasting),** mg/dL | | | | | | |
| Total cholesterol | 183 ± 24 | 179 ± 17 | 198 ± 46 | 192 ± 18 | 199 ± 27 | 204 ± 44 |
| LDL-cholesterol | 122 ± 24 | 117 ± 12 | 129 ± 38 | 131 ± 19 | 136 ± 24 | 137 ± 32 |
| HDL-cholesterol | 47 ± 7 | 47 ± 13 | 54 ± 16 | 49 ± 7 | 44 ± 11 | 46 ± 12 |
| Triglycerides | 92 ± 37 | 93 ± 24 | 106 ± 55 | 79 ± 16 | 127 ± 45 | 120 ± 42 |
| Insulin (µIU/mL) | 7.5 ± 3.4 | 9.6 ± 4.9 | 10.2 ± 2.6 | 12.1 ± 10.5 | 12.6 ± 6.2 | 11.4 ± 7.0 |
| Glucose | 100 ± 6 | 98 ± 9 | 101 ± 5 | 96 ± 10 | 100 ± 7 | 102 ± 13 |
| **Brachial artery blood pressure (fasting),** mmHg | | | | | | |
| Systolic | 130 ± 9 | 130 ± 11 | 127 ± 18 | 129 ± 4 | 134 ± 19 | 131 ± 10 |
| Diastolic | 76 ± 10 | 83 ± 11 | 81 ± 12 | 77 ± 8 | 85 ± 10 | 79 ± 5 |
| **Brachial artery endothelial function (fasting)** | | | | | | |
| Flow-mediated dilation, % | 6.6 ± 2.3 | 6.2 ± 2.5 | 6.5 ± 1.6 | 5.7 ± 2.1 | 5.5 ± 1.9 | 6.4 ± 3.6 |

Data are presented as mean ± SD, unless otherwise stated.

**LSM**: acute effect of low-spice meal (LSM) after 4-week exposure to low-spice diet (LSD)

**MSM**: acute effect of moderate-spice meal (MSM) after 4-week exposure to moderate-spice diet (MSD)

**HSM**: acute effect of high-spice meal (HSM) after 4-week exposure to high-spice diet (HSD)

**The Effect of Meals with Culinary Doses of Herbs/Spices on Postprandial Endothelial Function, Lipemia and Glycemia Following 4-week Exposure to Herb/Spice-Containing Diets: A Secondary Analysis of A Randomized, Crossover, Controlled-Feeding Study**

Damani *et al*. Online Supplemental Material

# Supplemental Table 2. Mean values for brachial artery endothelial function assessment by condition and timepoint^1^

|  | **Low Spice Meal** | | **Moderate Spice Meal** | | | **High Spice Meal** | | | ***P* values** | | | |
| --- | --- | --- | --- | --- | --- | --- | --- | --- | --- | --- | --- | --- |
| **Outcome^1^** | ***T*0** | ***T*120** | ***T*240** | ***T*0** | ***T*120** | ***T*240** | ***T*0** | ***T*120** | ***T*240** | **Condition** | **Time** | **Condition × Time** |
| Flow-mediated dilation (%) | 6.54 ± 0.38 | 7.29 ± 0.38 | 7.63 ± 0.38 | 6.67 ± 0.38 | 7.32 ± 0.38 | 7.82 ± 0.38 | 6.88 ± 0.38 | 7.72 ± 0.38 | 7.97 ± 0.38 | 0.293 | <.0001 | 0.995 |
| Resting artery diameter (mm) | 4.62 ± 0.12 | 4.61 ± 0.12 | 4.59 ± 0.12 | 4.70 ± 0.12 | 4.60 ± 0.12 | 4.60 ± 0.12 | 4.57 ± 0.12 | 4.60 ± 0.12 | 4.59 ± 0.12 | 0.297 | 0.448 | 0.477 |
| Peak artery diameter (mm) | 4.92 ± 0.12 | 4.94 ± 0.12 | 4.93 ± 0.12 | 5.00 ± 0.12 | 4.93 ± 0.12 | 4.96 ± 0.12 | 4.88 ± 0.12 | 4.95 ± 0.12 | 4.94 ± 0.12 | 0.416 | 0.968 | 0.488 |
| Peak artery dilation (mm) | 0.30 ± 0.02 | 0.33 ± 0.02 | 0.34 ± 0.02 | 0.30 ± 0.02 | 0.33 ± 0.02 | 0.36 ± 0.02 | 0.31 ± 0.02 | 0.35 ± 0.02 | 0.35 ± 0.02 | 0.343 | 0.0002 | 0.94 |
| Resting blood flow (mL/min)^2,3^ | 247 | 193 | 162 | 267 | 196 | 171 | 258 | 199 | 182 | 0.221 | <.0001 | 0.927 |
|  | (242, 338) | (182, 243) | (152, 204) | (261, 383) | (185, 268) | (162, 207) | (251, 350) | (187, 281) | (169, 253) |  |  |  |
| Peak blood flow (mL/min)^2,3^ | 1333 | 1269 | 1269 | 1379 | 1298 | 1312 | 1323 | 1284 | 1274 | 0.196 | 0.020 | 0.955 |
|  | (1266, 1555) | (1201, 1475) | (1204, 1469) | (1275, 1718) | (1233, 1560) | (1240, 1549) | (1254, 1573) | (1220, 1519) | (1204, 1495) |  |  |  |
| Reactive hyperemia (%)^3^ | 482 ± 34 | 597 ± 35 | 706 ± 34 | 455 ± 34 | 586 ± 35 | 686 ± 34 | 448 ± 35 | 584 ± 35 | 652 ± 35 | 0.389 | <.0001 | 0.964 |

^1^Data represent least squares means ± SEM, unless otherwise stated. PROC MIXED (SAS Institute, Cary, NC, USA) was used for analyses with participants nested within randomization sequence modeled as a repeated factor.

^2^Data that are non-normally distributed are presented as geometric mean (95% confidence interval).

LSM, n=43; MSM, n=43; HSM, n=43 unless otherwise stated.

^3^LSM, n=43; MSM, n=43; HSM, n=42

LSM: acute effect of low-spice meal (LSM) after 4-week exposure to low-spice diet (LSD)

MSM: acute effect of moderate-spice meal (MSM) after 4-week exposure to moderate-spice diet (MSD)

HSM: acute effect of high-spice meal (HSM) after 4-week exposure to high-spice diet (HSD)

**The Effect of Meals with Culinary Doses of Herbs/Spices on Postprandial Endothelial Function, Lipemia and Glycemia Following 4-week Exposure to Herb/Spice-Containing Diets: A Secondary Analysis of A Randomized, Crossover, Controlled-Feeding Study**

Damani *et al*. Online Supplemental Material

# Supplemental Table 3. Mean values for blood glucose, insulin, lipids, and lipoproteins by condition and timepoint^1^.

|  | **Low Spice Meal** | | | | | | **Moderate Spice Meal** | | | | | | **High Spice Meal** | | | | | | ***P*-values** | | |
| --- | --- | --- | --- | --- | --- | --- | --- | --- | --- | --- | --- | --- | --- | --- | --- | --- | --- | --- | --- | --- | --- |
| **Outcome (mg/dL)^1^** | ***T*0** | ***T*30** | ***T*60** | ***T*120** | ***T*180** | ***T*240** | ***T*0** | ***T*30** | ***T*60** | ***T*120** | ***T*180** | ***T*240** | ***T*0** | ***T*30** | ***T*60** | ***T*120** | ***T*180** | ***T*240** | **Condition** | **Time** | **Condition ×Time** |
| Glucose^2^ | 96 | 127 | 109 | 105 | 104 | 103 | 97 | 131 | 112 | 109 | 106 | 105 | 98 | 128 | 109 | 107 | 102 | 104 | 0.465 | <.0001 | 0.990 |
|  | (94, 99) | (120, 140) | (103, 122) | (102, 110) | (101, 110) | (99, 109) | (95, 100) | (125, 141) | (108, 120) | (105, 115) | (102, 112) | (102, 109) | (96, 100) | (120, 141) | (104, 121) | (104, 112) | (100, 108) | (101, 109) |  |  |  |
| Insulin^2^ (µIU/mL) | 9 | 89 | 61 | 40 | 35 | 22 | 9 | 103 | 69 | 51 | 34 | 25 | 8 | 98 | 69 | 46 | 35 | 23 | 0.058 | <.0001 | 0.806 |
|  | (8, 11) | (85, 140) | (59, 91) | (41, 60) | (34, 52) | (21, 34) | (8, 13) | (97, 150) | (67, 94) | (51, 72) | (34, 54) | (26, 40) | (8, 11) | (92, 155) | (68, 108) | (44, 68) | (33, 49) | (23, 36) |  |  |  |
| Triglycerides^2^ | 96 | 104 | 121 | 143 | 165 | 159 | 103 | 108 | 124 | 150 | 168 | 166 | 106 | 113 | 127 | 147 | 167 | 164 | 0.439 | <.0001 | 0.977 |
|  | (91, 116) | (98, 124) | (114, 141) | (136, 167) | (157, 196) | (151, 194) | (90, 140) | (98, 139) | (113, 154) | (139, 186) | (156, 207) | (154, 208) | (97, 134) | (104, 139) | (118, 155) | (136, 181) | (155, 208) | (154, 202) |  |  |  |
| Total Cholesterol | 189 ± 5 | 193 ± 5 | 192 ± 5 | 190 ± 5 | 188 ± 5 | 190 ± 5 | 186 ± 5 | 190 ± 5 | 189 ± 5 | 188 ± 5 | 186 ± 5 | 188 ± 5 | 192 ± 5 | 197 ± 5 | 194 ± 5 | 191 ± 5 | 190 ± 5 | 192 ± 5 | 0.049 | <.0001 | 0.987 |
| LDL-Cholesterol | 127 ± 4 | 127 ± 4 | 125 ± 4 | 122 ± 4 | 119 ± 4 | 120 ± 4 | 122 ± 4 | 123 ± 4 | 120 ± 4 | 118 ± 4 | 115 ± 4 | 116 ± 4 | 126 ± 4 | 128 ± 4 | 125 ± 4 | 122 ± 4 | 119 ± 4 | 120 ± 4 | 0.011 | <.0001 | 0.999 |
| HDL-Cholesterol^2^ | 45 | 45 | 45 | 43 | 42 | 43 | 44 | 45 | 44 | 43 | 42 | 42 | 43 | 44 | 43 | 42 | 41 | 42 | 0.632 | <.0001 | 0.999 |
|  | (43, 49) | (43, 50) | (42, 50) | (41, 48) | (40, 47) | (40, 48) | (42, 48) | (43, 50) | (42, 49) | (41, 47) | (40, 46) | (40, 47) | (41, 48) | (42, 49) | (41, 48) | (40, 47) | (39, 46) | (39, 46) |  |  |  |

^1^Data represent least squares means ± SEM, unless otherwise stated. PROC MIXED (SAS Institute, Cary, NC, USA) was used for analyses with participants nested within randomization sequence modeled as a repeated factor.

^2^Data that are non-normally distributed are presented as geometric mean (95% confidence interval).

LSM, *n*=43; MSM, *n*=42; HSM, *n*=41

LSM: acute effect of low-spice meal (LSM) after 4-week exposure to low-spice diet (LSD)

MSM: acute effect of moderate-spice meal (MSM) after 4-week exposure to moderate-spice diet (MSD)

HSM: acute effect of high-spice meal (HSM) after 4-week exposure to high-spice diet (HSD)

**The Effect of Meals with Culinary Doses of Herbs/Spices on Postprandial Endothelial Function, Lipemia and Glycemia Following 4-week Exposure to Herb/Spice-Containing Diets: A Secondary Analysis of A Randomized, Crossover, Controlled-Feeding Study**

Damani *et al*. Online Supplemental Material

# Supplemental Table 4. Relationship between the change in FMD (%) and change in triglycerides (mg/dL) by condition and timepoint^1^

|  |  | | **Change in FMD (%)** | | | | | | | |
| --- | --- | --- | --- | --- | --- | --- | --- | --- | --- | --- |
| **Change in triglycerides (mg/dL)** | | 120 minutes post meal intake | | | | | 240 minutes post meal intake | | | |
|  |  | Baseline | | LSM | MSM | HSM | Baseline | LSM | MSM | HSM |
|  |  | *r* = 0.08 | | *r* = −0.05 | *r* = −0.03 | *r* = −0.31 | *r* = −0.09 | *r* = 0.03 | *r* = −0.06 | *r* = 0.13 |
|  |  | *p* = 0.597 | | *p* = 0.732 | *p* = 0.840 | *p* = 0.049 | *p* = 0.583 | *p* = 0.874 | *p* = 0.726 | *p* = 0.423 |

^1^Data represent Pearson’s correlation coefficient r along with the p-value. PROC CORR (SAS Institute, Cary, NC, USA) was used to assess the relationship between the change in FMD (%) and triglycerides (mg/dL) from fasting state to 120 minutes and to 240 minutes

*Abbreviations*: Baseline: exposure to LSM at baseline without prior 4-week spice exposure; LSM: acute effect of low-spice meal (LSM) after 4-week exposure to low-spice diet; MSM: acute effect of moderate-spice meal (MSM) after 4-week exposure to moderate-spice diet; HSM: acute effect of high-spice meal (HSM) after 4-week exposure to high-spice diet

.

**The Effect of Meals with Culinary Doses of Herbs/Spices on Postprandial Endothelial Function, Lipemia and Glycemia Following 4-week Exposure to Herb/Spice-Containing Diets: A Secondary Analysis of A Randomized, Crossover, Controlled-Feeding Study**

Damani *et al*. Online Supplemental Material

# Supplemental Table 5. Between-person and within-person variance in the total AUC for FMD, blood glucose, insulin and triglycerides

| **Total Area Under the Curve (AUC)** | **Variance** | **Estimate** | **95% confidence interval** | | **Proportion of Total Variance (%)** |
| --- | --- | --- | --- | --- | --- |
|  |  |  | **Lower** | **Upper** |  |
| Brachial Artery FMD (% × min) | Between-person | 134973 | 85793 | 243038 | 63.8 |
|  | Within-person | 76637 | 57788 | 106541 | 36.2 |
| Glucose (mg/dL × min) | Between-person | 4218261 | 2469686 | 8791989 | 45.7 |
|  | Within-person | 5002483 | 3756503 | 6993792 | 54.3 |
| Insulin (µIU/mL × min) | Between-person | 48036762 | 31940450 | 80352774 | 84.1 |
|  | Within-person | 9083592 | 6827986 | 12681985 | 15.9 |
| Triglycerides (mg/dL × min) | Between-person | 198070000 | 130390000 | 336670000 | 78.9 |
|  | Within-person | 53096755 | 39904927 | 74148453 | 21.1 |

Between-person and within-person variation in the total AUC for FMD, blood glucose, insulin, and triglycerides was quantified using a linear mixed-effect model (PROC MIXED; SAS Institute, Cary, NC, USA) with randomization sequence and condition (LSM, MSM, or HSM) modeled as fixed effects and participant nested within randomization sequence modeled to account for the repeated-measures crossover design. The total AUC for the baseline visit where participants received the LSM was not included in the model as a covariate. The participant-level random intercept represented between-person variance, and the residual term represented within-person variance. The intraclass correlation coefficient was calculated as the random-intercept variance divided by total variance, indicating the proportion of variance attributable to between-person differences

Abbreviations: FMD, flow-mediated dilation; LSM: acute effect of low-spice meal (LSM) after 4-week exposure to low-spice diet; MSM: acute effect of moderate-spice meal (MSM) after 4-week exposure to moderate-spice diet; HSM: acute effect of high-spice meal (HSM) after 4-week exposure to high-spice diet

**The Effect of Meals with Culinary Doses of Herbs/Spices on Postprandial Endothelial Function, Lipemia and Glycemia Following 4-week Exposure to Herb/Spice-Containing Diets: A Secondary Analysis of A Randomized, Crossover, Controlled-Feeding Study**

Damani *et al*. Online Supplemental Material

# Supplemental Table 6. Multiple Linear Regression for Brachial Artery FMD: Total Area Under the Curve (AUC) (% × min)

| **Meal** | **Baseline Characteristics** | **Partial R² (%)** | **Beta Coefficient** | **95% CI** | ***p*-value** |
| --- | --- | --- | --- | --- | --- |
| **Condition** |  |  |  |  |  |
| Baseline Meal | Age (y) | 7.02 | −24.1 | −34.9, −13.4 | 0.011 |
|  | Sex | 6.61 | −432 | −688, −176 | 0.008 |
|  | BMI (kg/m^2^) | - | −12.0 | −46.7, 22.7 | 0.487 |
|  | Fasting FMD (%) | 54.76 | 161.2 | 119.6, 202.9 | <.0001 |
|  | MetS | 3.57 | 233.2 | 23.5, 442.8 | 0.037 |
| LSM after 4-week LSD | Age (y) | - | −10.2 | −21.4, 0.91 | 0.071 |
|  | Sex | - | −207 | −475, 61 | 0.126 |
|  | BMI (kg/m^2^) | - | −27.5 | −73.4, 18.3 | 0.232 |
|  | Fasting FMD (%) | 17.7 | 75.5 | 30.1, 121 | <.0001 |
|  | MetS | - | 69.5 | −158.7, 297.7 | 0.549 |
| MSM after 4-week MSD | Age (y) | - | −8.8 | −21, 3.4 | 0.153 |
|  | Sex | - | −248 | −543, 46.1 | 0.096 |
|  | BMI (kg/m^2^) | - | −17.2 | −57.2, 22.7 | 0.387 |
|  | Fasting FMD (%) | 25.3 | 97.3 | 49, 145 | <.0001 |
|  | MetS | - | −179 | −419, 60.9 | 0.05 |
| HSM after 4-week HSD | Age (y) | - | −9.4 | −23, 4.1 | 0.186 |
|  | Sex | - | −208 | −550, 133 | 0.224 |
|  | BMI (kg/m^2^) | - | −26.6 | −84.5, 31.2 | 0.357 |
|  | Fasting FMD (%) | 23.9 | 113 | 53, 174 | <.0001 |
|  | MetS | - | −193 | −527, 141 | 0.25 |

Partial R² is reported only for variables identified in the best-fit model from the stepwise, bidirectional model building. Partial R² quantifies the proportion of variance in the outcome explained by a predictor variable, after controlling for the effects of other predictors in the model. MetS: metabolic syndrome

*Abbreviations*: Baseline: exposure to LSM at baseline without prior 4-week spice exposure; LSM: acute effect of low-spice meal (LSM) after 4-week exposure to low-spice diet; MSM: acute effect of moderate-spice meal (MSM) after 4-week exposure to moderate-spice diet; HSM: acute effect of high-spice meal (HSM) after 4-week exposure to high-spice diet

**The Effect of Meals with Culinary Doses of Herbs/Spices on Postprandial Endothelial Function, Lipemia and Glycemia Following 4-week Exposure to Herb/Spice-Containing Diets: A Secondary Analysis of A Randomized, Crossover, Controlled-Feeding Study**

Damani *et al*. Online Supplemental Material

# Supplemental Table 7. Multiple Linear Regression for Postprandial Glucose: Total Area Under the Curve (AUC) (mg/dL × min)

| **Meal** | **Baseline Characteristics** | **Partial R² (%)** | **Beta Coefficient** | **95% CI** | ***p-*value** |
| --- | --- | --- | --- | --- | --- |
| **Condition** |  |  |  |  |  |
| Baseline Meal | Age (y) | - | 34.2 | −50, 118 | 0.415 |
|  | Sex | - | −818 | −3037, 1401 | 0.459 |
|  | BMI (kg/m^2^) | - | −135 | −411, 141 | 0.326 |
|  | Fasting Glucose (mg/dL) | 31.1 | 222 | 118, 327 | <.0001 |
|  | MetS | - | 170 | −1544, 1883 | 0.842 |
| LSM after 4-week LSD | Age (y) | - | 21 | −49, 90 | 0.553 |
|  | Sex | - | −360 | −2162, 1443 | 0.688 |
|  | BMI (kg/m^2^) | - | −196 | −421, 29 | 0.086 |
|  | Fasting Glucose (mg/dL) | 35.6 | 221 | 120, 323 | <.0001 |
|  | MetS | - | 348 | −1047, 1743 | 0.616 |
| MSM after 4-week MSD | Age (y) | - | 12 | −115, 113 | 0.832 |
|  | Sex | - | 1012 | −1837, 4013 | 0.475 |
|  | BMI (kg/m^2^) | - | −143 | −533, 222 | 0.434 |
|  | Fasting Glucose (mg/dL) | 11.8 | 133 | 15, 251 | 0.027 |
|  | MetS | - | 1314 | −944, 3571 | 0.245 |
| HSM after 4-week HSD | Age (y) | - | −8.2 | −130, 114 | 0.892 |
|  | Sex | - | −731 | −3857, 2395 | 0.638 |
|  | BMI (kg/m^2^) | - | −213 | −606, 181 | 0.28 |
|  | Fasting Glucose (mg/dL) | - | 58 | −95, 210 | 0.448 |
|  | MetS | - | 1351 | −1092, 3793 | 0.27 |

Partial R² is reported only for variables identified in the best-fit model from the stepwise, bidirectional model building. Partial R² quantifies the proportion of variance in the outcome explained by a predictor variable, after controlling for the effects of other predictors in the model. MetS: metabolic syndrome

*Abbreviations*: Baseline: exposure to LSM at baseline without prior 4-week spice exposure; LSM: acute effect of low-spice meal (LSM) after 4-week exposure to low-spice diet; MSM: acute effect of moderate-spice meal (MSM) after 4-week exposure to moderate-spice diet; HSM: acute effect of high-spice meal (HSM) after 4-week exposure to high-spice diet

**The Effect of Meals with Culinary Doses of Herbs/Spices on Postprandial Endothelial Function, Lipemia and Glycemia Following 4-week Exposure to Herb/Spice-Containing Diets: A Secondary Analysis of A Randomized, Crossover, Controlled-Feeding Study**

Damani *et al*. Online Supplemental Material

# Supplemental Table 8. Multiple Linear Regression for Postprandial Insulin: Total Area Under the Curve (AUC) (µIU/mL × min)

| **Meal** | **Baseline Characteristics** | **Partial R² (%)** | **Beta Coefficient** | **95% CI** | ***p-*value** |
| --- | --- | --- | --- | --- | --- |
| **Condition** |  |  |  |  |  |
| Baseline Meal | Age (y) | - | 100 | −123, 323 | 0.369 |
|  | Sex | - | −1327.1 | −7013, 4359 | 0.639 |
|  | BMI (kg/m^2^) | - | −747.5 | −1540, 45 | 0.064 |
|  | Fasting Insulin (µIU/mL) | 35.5 | 1088.9 | 669, 1509 | <.0001 |
|  | MetS | - | −787.6 | −5330, 3755 | 0.727 |
| LSM after 4-week LSD | Age (y) | - | −20 | −262, 222 | 0.866 |
|  | Sex | - | −1631 | −7678, 4416 | 0.588 |
|  | BMI (kg/m^2^) | - | −799 | −1647, 49 | 0.064 |
|  | Fasting Insulin (µIU/mL) | 15.7 | 718 | 257, 1179 | 0.003 |
|  | MetS | - | 584 | −4360, 5528 | 0.812 |
| MSM after 4-week MSD | Age (y) | - | 5 | −252, 262 | 0.967 |
|  | Sex | - | −1570 | −7905, 4766 | 0.618 |
|  | BMI (kg/m^2^) | - | −511 | −1429, 406 | 0.266 |
|  | Fasting Insulin (µIU/mL) | 29.9 | 1026 | 542, 1510 | <.0001 |
|  | MetS | - | −1627 | −6819, 3564 | 0.529 |
| HSM after 4-week HSD | Age (y) |  | 66 | −148, 281 | 0.534 |
|  | Sex |  | −1178 | −6485, 4129 | 0.656 |
|  | BMI (kg/m^2^) |  | −646 | −1394, 102 | 0.089 |
|  | Fasting Insulin (µIU/mL) | 32.8 | 933 | 528, 1339 | <.0001 |
|  | MetS |  | 558 | −3736, 4852 | 0.794 |

Partial R² is reported only for variables identified in the best-fit model from the stepwise, bidirectional model building. Partial R² quantifies the proportion of variance in the outcome explained by a predictor variable, after controlling for the effects of other predictors in the model. MetS: metabolic syndrome

*Abbreviations*: Baseline: exposure to LSM at baseline without prior 4-week spice exposure; LSM: acute effect of low-spice meal (LSM) after 4-week exposure to low-spice diet; MSM: acute effect of moderate-spice meal (MSM) after 4-week exposure to moderate-spice diet; HSM: acute effect of high-spice meal (HSM) after 4-week exposure to high-spice diet

**The Effect of Meals with Culinary Doses of Herbs/Spices on Postprandial Endothelial Function, Lipemia and Glycemia Following 4-week Exposure to Herb/Spice-Containing Diets: A Secondary Analysis of A Randomized, Crossover, Controlled-Feeding Study**

Damani *et al*. Online Supplemental Material

# Supplemental Table 9. Multiple Linear Regression for Postprandial Triglycerides: Total Area Under the Curve (AUC) (mg/dL × min)

| **Meal** | **Baseline Characteristics** | **Partial R² (%)** | **Beta Coefficient** | **95% CI** | ***p-*value** |
| --- | --- | --- | --- | --- | --- |
| **Condition** |  |  |  |  |  |
| Baseline Meal | Age (y) |  | −132 | −335, 71 | 0.195 |
|  | Sex |  | −421 | −5521, 4679 | 0.868 |
|  | BMI (kg/m^2^) |  | 614 | −55, 1283 | 0.071 |
|  | Fasting Triglycerides (mg/dL) | 82.1 | 297 | 242, 352 | <.0001 |
|  | MetS |  | 1042 | −3617, 5701 | 0.653 |
| LSM after 4-week LSD | Age (y) |  | 226 | −260, 714 | 0.351 |
|  | Sex |  | 6386 | −5496, 18268 | 0.283 |
|  | BMI (kg/m^2^) |  | −35 | −1620, 1549 | 0.964 |
|  | Fasting Triglycerides (mg/dL) | 41.1 | 235 | 102, 368 | 0.001 |
|  | MetS |  | 965 | −10177, 12109 | 0.861 |
| MSM after 4-week MSD | Age (y) |  | 269 | −98, 635 | 0.146 |
|  | Sex |  | 5361 | −3432, 14154 | 0.224 |
|  | BMI (kg/m^2^) |  | 411 | −803, 1625 | 0.497 |
|  | Fasting Triglycerides (mg/dL) | 32.3 | 144 | 45, 244 | 0.006 |
|  | MetS |  | −401 | −8871, 8070 | 0.924 |
| HSM after 4-week HSD | Age (y) |  | 286 | −311, 882 | 0.338 |
|  | Sex |  | 9117 | −5329, 23563 | 0.209 |
|  | BMI (kg/m^2^) |  | 204 | −1728, 2137 | 0.832 |
|  | Fasting Triglycerides (mg/dL) | 24.1 | 178 | 16, 340 | 0.032 |
|  | MetS |  | 1397 | −12206, 14999 | 0.836 |

Partial R² is reported only for variables identified in the best-fit model from the stepwise, bidirectional model building. Partial R² quantifies the proportion of variance in the outcome explained by a predictor variable, after controlling for the effects of other predictors in the model. MetS: metabolic syndrome

*Abbreviations*: Baseline: exposure to LSM at baseline without prior 4-week spice exposure; LSM: acute effect of low-spice meal (LSM) after 4-week exposure to low-spice diet; MSM: acute effect of moderate-spice meal (MSM) after 4-week exposure to moderate-spice diet; HSM: acute effect of high-spice meal (HSM) after 4-week exposure to high-spice diet
